# Post-Traumatic Cerebral Infarction: A Narrative Review of Pathophysiology, Diagnosis, and Treatment

**DOI:** 10.3390/neurolint16010006

**Published:** 2024-01-04

**Authors:** Roy A. Poblete, Charlotte Zhong, Anish Patel, Grace Kuo, Philip Y. Sun, Jiayu Xiao, Zhaoyang Fan, Nerses Sanossian, Amytis Towfighi, Patrick D. Lyden

**Affiliations:** 1 Keck School of Medicine, The University of Southern California, Los Angeles, CA 90033, USA; charlotte.zhong@gmail.com (C.Z.); anishp88@gmail.com (A.P.); grace.kuo@med.usc.edu (G.K.); jiayu.xiao2@med.usc.edu (J.X.); zhaoyang.fan@med.usc.edu (Z.F.); nsanossi@usc.edu (N.S.); amytis.towfighi@med.usc.edu (A.T.); plyden@usc.edu (P.D.L.); 2 David Geffen School of Medicine, University of California Los Angeles, Los Angeles, CA 90095, USA; philipsun@mednet.ucla.edu; 3 Zilkha Neurogenetic Institute, Keck School of Medicine, The University of Southern California, Los Angeles, CA 90033, USA

**Keywords:** traumatic brain injury, stroke, cervical artery dissection, cerebral venous thrombosis, vasospasm

## Abstract

Traumatic brain injury (TBI) is a common diagnosis requiring acute hospitalization. Long-term, TBI is a significant source of health and socioeconomic impact in the United States and globally. The goal of clinicians who manage TBI is to prevent secondary brain injury. In this population, post-traumatic cerebral infarction (PTCI) acutely after TBI is an important but under-recognized complication that is associated with negative functional outcomes. In this comprehensive review, we describe the incidence and pathophysiology of PTCI. We then discuss the diagnostic and treatment approaches for the most common etiologies of isolated PTCI, including brain herniation syndromes, cervical artery dissection, venous thrombosis, and post-traumatic vasospasm. In addition to these mechanisms, hypercoagulability and microcirculatory failure can also exacerbate ischemia. We aim to highlight the importance of this condition and future clinical research needs with the goal of improving patient outcomes after TBI.

## 1. Introduction

### 1.1. The Health and Socioeconomic Impact of Traumatic Brain Injury

Traumatic brain injury (TBI) is a significant cause of death and long-term disability in the United States (US) and worldwide. According to the US Centers for Disease Control and Prevention, TBI results in 2.9 million emergency room visits, 288,000 annual hospitalizations, and 57,000 TBI-related deaths [1]. In addition to high mortality rates, TBI survivors often suffer from long-term functional disability with cognitive, emotional, behavioral, and physical impairments [2]. The impact both on the individual and society at large is significant. The cumulative lifetime medical costs of TBI are estimated to be USD 76.5 billion nationwide [3,4]. In addition to modifying systems of care, advancements in medical knowledge are needed to improve outcomes in this population. 

### 1.2. Secondary Brain Injury in Traumatic Brain Injury

Primary TBI prevention has the greatest impact on the overall burden of disease; however, limiting secondary injury after TBI is a clinical priority. After the initial impact or acceleration–deceleration injury, multiple pathophysiologic mechanisms lead to secondary brain injury, including delayed hemorrhagic expansion, ischemic injury and microthrombi formation, neuroinflammation, oxidative stress, metabolic dysfunction, and cerebral edema. Neuroinflammation, which begins early after TBI, plays a central role in mediating secondary neuronal death [5]. This process involves a cascade of reactions leading to excitotoxicity from the activation of glutamate and calcium receptors, phospholipid membrane breakdown, and microglial activation mediated by chemokines and cytokines that remain elevated several months after TBI [6,7,8,9]. Inflammation also promotes blood–brain barrier (BBB) permeability, resulting in a low-resistance pathway for pro-inflammatory cytokines, macrophages, neutrophils, and water into brain parenchyma [10]. The resulting neuronal injury and cerebral edema can lead to irreversible brain injury and life-threatening herniation syndromes. These deleterious effects can be minimized with appropriate post-TBI monitoring and care.

### 1.3. Post-Traumatic Cerebral Infarction: An Important Cause of Secondary Brain Injury

Given the morbidity and mortality associated with TBI, it is important to recognize common causes of secondary brain injury. Although, individually, acute ischemic stroke (AIS) and TBI are extensively studied and described in the literature, less is known about the development and treatment of post-traumatic cerebral infarction (PTCI). The following review provides a clinically relevant description of the relevance, incidence, pathophysiology, and management of AIS occurring acutely after TBI. In this review, we also highlight potential future treatment and research directions aiming to limit the negative impact of PTCI. To our knowledge, there is no recent comprehensive review on this topic. 

Delayed cerebral ischemia is a feared complication of TBI that worsens secondary brain injury and is potentially preventable. PTCI is not fully characterized; however, observational studies demonstrate its impact on patient outcomes. The true incidence of PTCI is unknown but is reported to be 2.1–12% based on retrospective reviews [11,12,13], often occurring within the first two weeks of TBI [13]. Differences in the reported incidence of PTCI are likely due to heterogenous patient populations and diagnostic criteria. Research has consistently demonstrated an association between PTCI and increased mortality [11,12,13]. In a large US secondary database analysis by Kowalski et al., AIS after TBI was found to result in poor outcomes by multiple functional measures, including a 13.3-point reduction in Functional Independence Measure total score, a 1.9-point increase in Disability Rating Scale score, and an 18-day increase in post-TBI amnesia duration [11].

The largest identified risk factors for PTCI are a low Glasgow Coma Scale (GCS) score on presentation, brain herniation, a need for decompressive craniectomy (DC), and cervical artery (carotid or vertebral artery) dissection (CAD) [11,12,13]. Although age is a significant risk factor for spontaneous AIS, its influence on PTCI incidence is unknown. Unlike AIS, TBI affects all age groups, with the majority of PTCI occurring in those <40 years old [11]. The prevention of AIS after TBI is especially important given the greater lifetime burden of post-TBI disability in younger patients. 

## 2. Pathophysiology

Many subclinical events increase PTCI risk, both at the macroscopic and the cellular level. As a final common pathway, mismatch between metabolic demand and oxygen delivery results in ischemic injury, and if sustained, results in neuronal death. Metabolic demand can be reduced through appropriate sedation and analgesia and by treating hypermetabolic states such as seizures and fever. Oxygen delivery can be optimized by the frequent monitoring of oxygen saturation, partial pressure of blood oxygen, blood pressure, and cerebral perfusion pressure (CPP). Avoidance of hypotension is critical as it exacerbates ischemic injury. Among pre-hospital TBI patients, each 10-point decrease in systolic pressure below the normal range is associated with an 18.8% increase in the adjusted odds of death [14], while the presence of both hypotension and hypoxia predict a significantly higher mortality than either alone [15].

**Figure 1:**
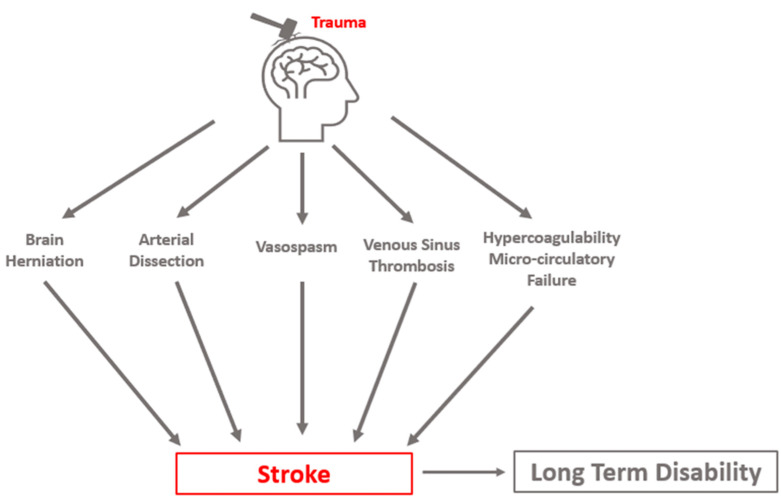
Conceptual schematic of primary etiologies of post-traumatic cerebral infarction.

Three distinct clinical syndromes are most causative of clinically relevant PTCI: cerebral herniation, CAD, and post-traumatic vasospasm (PTV, VSP). Stroke may occur in a delayed fashion, highlighting the importance of the early identification and appropriate management of risk factors. Cerebral infarct due to sinus thrombosis can occur but is a less common cause of PTCI. Additional sequelae of TBI, such as coagulopathy and microcirculatory failure, likely exacerbate delayed cerebral ischemia from these mechanisms (Figure 1).

### 2.1. Hypercoagulability after Traumatic Brain Injury

Coagulopathy after polytrauma is well described, but also occurs in 36–45% of isolated moderate-to-severe TBI patients [16,17]. As in polytrauma, both hypocoagulable and hypercoagulable states can exist. While hypocoagulability is implicated in hemorrhagic expansion after TBI, hypercoagulability results in an increased risk for PTCI. Proposed causes of hypercoagulability include platelet hyperactivity induced by platelet-activating factor (PAF), increased expression of tissue factor, and early disseminated intravascular coagulation [18,19]. A review of coagulopathies after TBI can be found in articles by Zhang et al. [18] and Laroche et al. [19].

### 2.2. Brain Herniation

Brain herniation is the most feared complication after TBI, as it is rapidly progressive and potentially fatal. The likelihood of herniation increases with TBI severity. In a recent study of severe TBI, 44% of patients had clinical signs of herniation on presentation [20]. When the clinical exam is limited, herniation might only be appreciated with early neuroimaging performed within 24–48 h of injury [21]. Progression to herniation is associated with mortality rates exceeding 80% [22]. As a predictor of delayed complications and less favorable outcomes, the presence of herniation can have a significant impact on patient management and goals of care.

In most cases, brain herniation is associated with elevated intracranial pressure (ICP) secondary to mass lesions from hemorrhage and cerebral edema [23]. Described by the Monro–Kellie doctrine, when compliance of the intracranial compartments (blood, brain, cerebrospinal fluid) is exceeded, herniation can potentially occur as the brain shifts to regions of lower pressure. If intracranial hypertension is not quickly treated, irreversible injury occurs.

**Figure 2:**
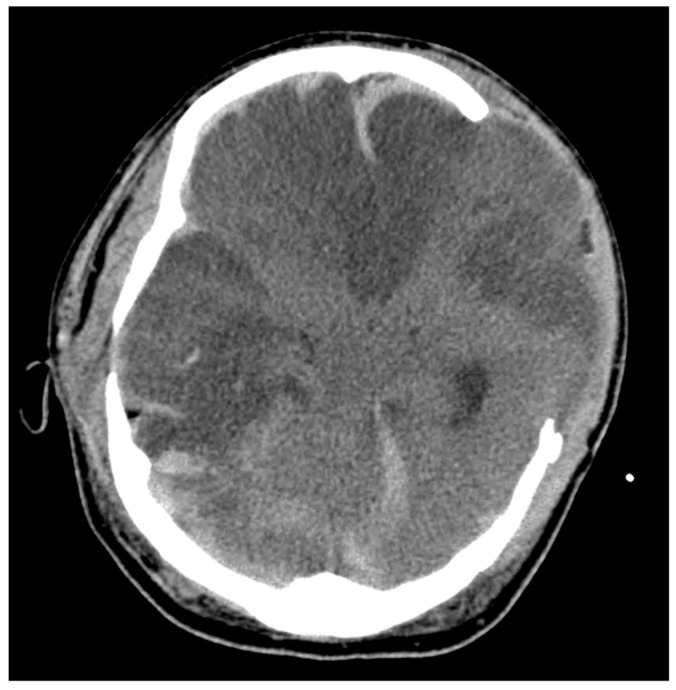
Illustrative case. A patient with TBI after a skateboarding accident requiring surgical decompression with a delayed head CT demonstrating loss of gray–white differentiation, midline shift, and extensive areas of hypodensity with notable subfalcine herniation leading to bilateral anterior cerebral artery strokes.

Brain herniation after TBI can lead to secondary ischemia through several mechanisms. Large territory infarcts are associated with specific herniation syndromes as displaced brain tissue mechanically compresses adjacent vessels. In transtentorial (uncal) herniation, descending supratentorial tissue compresses the posterior cerebral artery (PCA) and its branches, including the calcarine branch, leading to PCA infarction in the inferior temporal and occipital lobe ipsilateral to the herniation. In patients with a reliable clinical exam, a rapid change in level of consciousness and contralateral hemiparesis or posturing is observed. In intubated and sedated patients, pupillary dilation and sluggish reactivity are often the first observable signs [24,25]. In subfalcine herniation, the anterior cerebral artery (ACA) on the ipsilateral or contralateral side is compressed as the cingulate gyrus migrates across the falx (Figure 2). Clinically, this can manifest as cognitive dysfunction, a reduced level of consciousness, and predominant leg weakness. Lesions causing increased pressure in the posterior fossa are of special concern. Herniation of the cerebellar tonsils can compress the vertebral and posterior inferior cerebellar arteries, potentially causing PTCI in the brainstem and other areas within the posterior circulation.

Brain herniation can also signal an increased susceptibility to diffuse cerebral ischemia. Since ICP and CPP are inversely related, as ICP increases, CPP decreases. If vascular compliance is exceeded and further vasodilation is not possible, elevated ICPs result in ischemia [21]. Cerebral autoregulation, which normally maintains cerebral blood flow (CBF) despite fluctuation in CPP, is impaired in 49–87% of moderate-to-severe TBI patients and increases the brain’s susceptibility to ischemic injury from intracranial hypertension [26,27,28].

### 2.3. Cervical Artery Dissection

Cervical artery dissection is a relatively uncommon complication of TBI. It is observed in approximately 2% of moderate-to-severe TBI survivors, with carotid and vertebral artery dissections being equally likely [11]. The true prevalence of CAD is likely higher as most patients are asymptomatic; however, when present, it is widely recognized as an important cause of preventable PTCI. Moderate-to-severe TBI survivors with traumatic CAD have a 9.4% incidence of PTCI and are associated with worse functional and cognitive outcomes [11]. The mortality rate from traumatic CAD is difficult to ascertain as patients with severe brain injury often die before a diagnosis is made and likely have multiple factors contributing to death [29]. Given the association between CAD and poor outcomes, timely recognition and treatment are critical.

**Figure 3:**
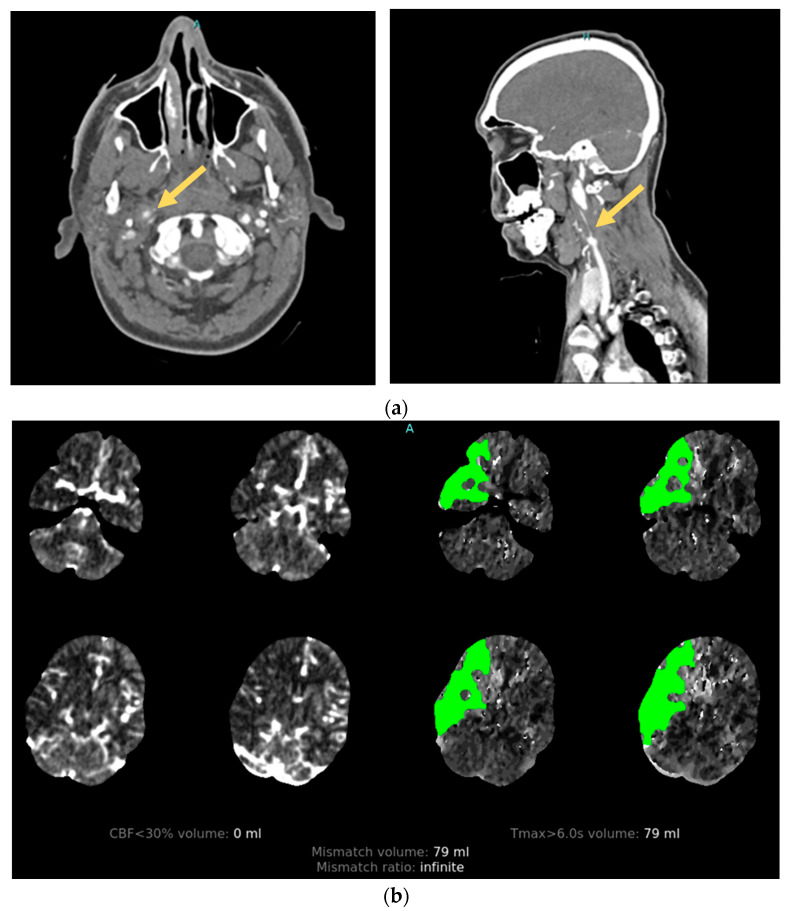
Illustrative case. A patient with traumatic cervical artery dissection after a motor vehicle accident. (**a**) CT angiogram shows right internal carotid dissection accompanied by pseudoaneurysm formation (arrows). (**b**) CT perfusion demonstrates a large area of delayed filling in the distribution of the dissected vessel.

High-impact injuries are strongly associated with CAD. Their development is attributed to rapid acceleration–deceleration leading to hyperextension and rotation of the neck, the stretching of vessels over adjacent vertebral structures, and the production of a tear in the intimal layer of the artery [30]. After endothelial injury, circulating blood under arterial pressure penetrates the damaged vessel wall, separating the intima and media layers. The resulting intramural hematoma can create a combination of macroscopic vascular defects that include vessel occlusion, stenosis, and pseudoaneurysm that can be seen on angiographic imaging [31]. Damage to the arterial intima also exposes subendothelial collagen to the intravascular space, which acts to trigger the intrinsic coagulation pathway, leading to platelet aggregation and activation with thrombi formation [32]. The majority of AIS secondary to CAD is subsequently caused by thromboembolism from the dissection site [33] (Figure 3). Less commonly, ischemic stroke can occur from hypoperfusion injury distal to occlusion or flow-limiting stenosis in patients with relative hypotension [33].

### 2.4. Post-Traumatic Vasospasm

Vasospasm can have a significant impact on functional outcomes after TBI. It is extensively studied in the context of aneurysmal subarachnoid hemorrhage (aSAH), but fewer data are available regarding the epidemiology and pathophysiology of PTV. Although PTV and VSP after aSAH likely share many similarities, a better understanding of the unique characteristics of PTV contributing to secondary brain injury is needed. The consequences of PTV are significant. In an observational study of pediatric patients, a good neurologic outcome (defined as a 30-day GCS score of ≥4) after moderate-to-severe TBI was seen in 76% of patients without VSP, compared to 40% in those with VSP [34]. In adults, PTV is associated with an increased likelihood of unfavorable clinical outcome compared to those without (29% vs. 15%) [35]. It is unknown whether PTV mediates poor outcomes or whether it is a biomarker for disease severity underlying poor outcomes.

PTV is not routinely assessed unless clinical signs or symptoms suggest its presence, leading to underdiagnosis. In clinical practice, VSP screening is considered when a focal neurologic deficit or mental status change is not explained by neuroimaging, electroencephalogram (EEG), or comorbid condition. VSP is also considered with new radiographic evidence of ischemia not caused by the primary injury. Early angiographic studies reported PTV incidence rates between 5% and 19% [34,36]; however, more recent neurosonography and imaging studies suggest a higher rate, ranging from 27% to 63% in adults and approximately 36% in the pediatric population [34,36,37]. Risk factors for PTV are not fully established, but age < 30 years, presence of SAH, and admission GCS ≤ 8 appear to be the strongest predictors [38]. Although most frequently associated with subarachnoid hemorrhage, PTV is known to occur in TBI even in the absence of SAH, including in blast injuries [39].

The timing of PTV onset and its natural course is thought to be different than that of aSAH. VSP typically occurs between days 4 and 15 following aSAH, rarely appearing prior to post-bleed day 3 [36,40]. In contrast, observational studies have suggested that 25% of VSP secondary to traumatic SAH occurs by day 3, and often within the first 2 days of injury [38]. When it does occur, the duration of VSP is often shorter than that of aSAH. In most cases, VSP largely resolves within 14 days of TBI, although cases lasting up to 30 days have been reported [38]. When PTV occurs in the absence of subarachnoid hemorrhage, VSP may have a very brief duration [35].

The proposed primary mechanism of PTV development includes the mechanical stretching and irritation of the blood vessel, resulting in inflammation and arterial smooth muscle spasm. The anatomic region of greatest hemorrhage density often correlates with the location of the spasm [35]. Secondary processes also contribute to vessel narrowing and spasm. These include the chronic depolarization of vascular smooth muscle due to reduced potassium channel activity, the breakdown of blood products resulting in endothelin release and reduced availability of nitric oxide, free radical formation, cyclic GMP depletion of vascular smooth muscle, and potentiation of prostaglandin-induced vasoconstriction [41,42,43]. Whether VSP becomes symptomatic depends on several clinical factors. Systemic conditions like hypoxia or hypotension, along with reduced CBF from elevated ICP, may contribute to impaired cerebral perfusion and oxygen delivery, resulting in symptomatic PTCI.

### 2.5. Cerebral Venous Thrombosis

Cerebral venous sinus thrombosis (CVST) can cause stroke by impeding venous flow. Its true incidence after TBI is unknown, but it may be more commonly identified with the increased use of advanced brain and vascular imaging techniques. Although post-TBI CVST often follows a benign course, intracerebral complications such as PTCI, hemorrhage, and cerebral edema can occur and are associated with increased mortality [44,45]. The primary risk factor for acute CVST is skull fracture resulting in sinus injury [46], with CVST rarely occurring without an overlying skull fracture. The general hypercoagulability observed after trauma is likely an exacerbating factor. Pathologic mechanisms leading to PTCI include increased ICP, reduced capillary perfusion, and peri-hematomal ischemia in the presence of intracerebral hemorrhage [47].

### 2.6. Microthrombosis and Microcirculatory Failure

Ischemic injury burden after TBI may not be grossly appreciated. Microthrombosis, an emerging pathophysiologic concept that occurs in several types of critical illness, is thought to worsen ischemic burden and secondary brain injury in small, distal cerebral vessels. Although the mechanisms are not entirely understood, inflammatory and thrombotic processes appear closely linked [48]. In vitro studies have demonstrated that TBI can trigger the aggregation of activated platelets, resulting in reduced peri-lesional blood flow [49,50]. Thrombus formation may be independent of disease severity and injury pattern, making it difficult to predict and diagnose in clinical practice [51,52]. Its clinical relevance is not established, with a need to further understand its pathophysiology and importance in TBI.

## 3. Diagnosis

### 3.1. Clinical Diagnosis of Acute Ischemic Stroke

Post-traumatic cerebral infarction is diagnosed using similar radiographic modalities used in spontaneous AIS, primarily non-contrast computed tomography (CT) and magnetic resonance imaging (MRI) of the brain. Although imaging is essential for anatomic characterization and definitive diagnosis, AIS is first clinically suspected due to either an acute change in neurologic status or failure to neurologically improve. Asymmetry in the motor exam may or may not be apparent. An accurate neurologic examination is often confounded by a low GCS score secondary to several factors commonly present in critically ill patients: traumatic, toxic, and metabolic encephalopathy, use of sedatives and analgesics, and intensive care unit (ICU) delirium. Physical barriers, including behavioral restraints, lines and drains, and bedrest orders also limit comprehensive neurologic testing and should be minimized when safe. Diagnostic modalities, including clinical considerations and limitations, are summarized in Table 1.

### 3.2. CT and MRI Neuroimaging Modalities

Current guidelines recommend non-contrast multidetector CT (MDCT) of the brain for the routine initial imaging after TBI [53,54]. MDCT is widely available and can quickly diagnose bony fractures, foreign objects, and acute intracranial hemorrhage (ICH) to identify patients requiring urgent interventions. AIS is identified on MDCT or MRI by the presence of cytotoxic edema in a vascular territory. With MDCT, the sensitivity of detecting AIS is influenced by the duration of ischemia and the size and location of the lesion. For large-territory ischemia, hyperacute findings of hypoattenuation of deep nuclei and loss of cortical grey–white differentiation can be seen within hours [55]. Over hours to days, hypoattenuation from edema becomes more pronounced and is often accompanied with increasing mass effect, especially in larger stroke. 

The use of MRI in the initial evaluation of TBI is limited by its availability and longer acquisition times; however, in select patients, its increased sensitivity for ischemia is important in diagnosing PTCI. MRI is often triggered when the neurologic exam or recovery is less than expected based on MDCT or early neuroimaging. MR characteristics are similar to AIS without TBI. During the first week (acute period), the infarcted brain demonstrates a high diffusion-weighted imaging (DWI) signal and corresponding low apparent diffusion coefficient (ADC) signal, with ADC typically fully normalizing between 10 and 15 days after AIS [56]. Ischemia involving the cortex commonly demonstrates a high T1 and T2 signal with an increased gradient echo and susceptibility-weighted imaging signal in the subacute-to-chronic phase [56]. This reflects cortical necrosis following ischemic injury rather than trauma-related hemorrhagic contusion. 

Differentiating PTCI from trauma-related contusion, cerebral edema, surgical complications, or expected post-surgical changes is a diagnostic challenge. The vascular distribution of a brain lesion is helpful and requires a basic understanding of neuroanatomy. The timing and progression of imaged lesions is also useful: contusions are unlikely to become newly apparent several days after TBI, and vasogenic edema from an ICH might be restricted to the perihematomal region. As discussed, brain herniation following TBI is strongly associated with PTCI and leads to AIS in specific vascular distributions [12,57]. Uncal herniation is characterized by the displacement of the mesial temporal lobe across and below the tentorium into the ambient cistern. This results in an ipsilateral occipital lobe infarct secondary to PCA compression [58]. Subfalcine herniation occurs when the cingulate gyrus of the frontal lobe is displaced across the anterior falx cerebri, leading to the impingement of the ACA and subsequent stroke [59]. The resulting ACA infarct can occur on the ipsilateral or contralateral side, depending on which ACA vessel is most severely compressed. 

Infarcts secondary to CAD are most often seen as embolic-appearing AIS within a vascular distribution distal to the dissected vessel. After blunt-force injury, this can occur in the internal carotid arteries supplying the anterior circulation, or the vertebral arteries supplying the posterior circulation. MR infarct patterns from PTV have not been fully characterized; however, in aSAH, they are heterogenous. In one observational study of 117 patients with aSAH, the following MRI patterns were seen: territorial (27%), lacunar (20%), watershed infarct (26%), and band-like cortical infarcts (7%) [60]. From our experience, PTCI due to VSP more commonly results in small-to-medium-size infarcts rather than large vascular territorial ones, and therefore may be associated with only subtle physical exam findings.

### 3.3. Vessel Imaging

#### 3.3.1. Angiography

**Figure 4:**
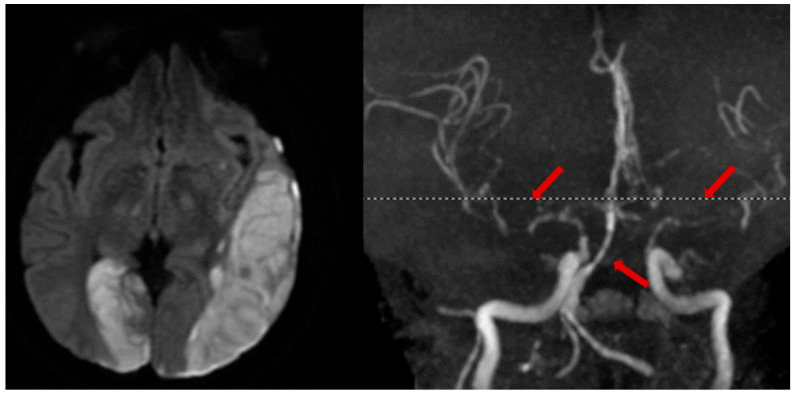
Illustrative case. A patient with a traumatic left subdural hematoma after assault, with MRA showing severe vasospasm in the bilateral anterior and middle cerebral arteries, mid-to-distal basilar, and posterior cerebral arteries (**right**, arrows), leading to acute ischemic strokes in the left MCA and right PCA territories (**left**).

In select cases of suspected or high risk of cervical artery injury, a contrast CT angiogram (CTA) of neck vessels is recommended. A CTA of the head and neck can be rapidly conducted and carries little additional risk. Although a clinical concern over contrast-induced nephropathy exists, the incidence of renal sequelae is low when performed in emergency AIS protocols [61]. Alternatively, an MR angiogram (MRA) with MRI for stroke evaluation can be performed (Figure 4).

No current guidelines are widely used to outline when a CTA or MRA should be performed; however, this is mostly dictated by institutional TBI protocols. Common indications include the presence of a cervical bone fracture traversing the carotid canal or through the expected course of the vertebral artery, the presence of penetrating neck injuries, or when ischemic or hemorrhagic lesions seen on neuroimaging are atypical for TBI [53,62,63]. In this setting, angiography with CTA or MRA might identify TBI mimickers, including ICH from vascular malformation, ischemic stroke with hemorrhagic transformation, early PTV, or ischemic and hemorrhagic venous stroke from CVST. CT venography may be of special value in patients with skull fracture and clinical suspicion of CVST [46]. In a recent retrospective review, routine CTA for TBI did not commonly change clinical management [64]; therefore, it should only be used in select cases.

#### 3.3.2. MR Vessel Wall Imaging: An Emerging Diagnostic Tool

MR vessel wall imaging (VWI) directly visualizes the vessel wall by suppressing blood signals in the artery lumen, allowing for the assessment of vessel wall pathologies that might lead to ischemic infarct. Compared to standard angiographic imaging, MR VWI can differentiate underlying pathologies, accurately evaluate the severity of vessel disease, and monitor its progression. It may be especially useful in evaluating arterial dissection and venous thrombosis. The diagnostic value in arterial dissection has been demonstrated in both cervical and intracranial arteries [65,66,67]. Classic MR VWI presentations of dissection include the presence of a double lumen, “string” sign, or a crescent-shaped intramural hematoma. Venous thrombosis appears as heterogeneous signal intensity within the dark lumen of the venous sinus. MR VWI has shown excellent diagnostic value in acute CVST [68,69], and can be used to predict treatment response to endovascular therapy [70]. Given that this diagnostic tool is non-invasive, radiation-free, and highly accurate, MR VWI may be optimal for the longitudinal evaluation of post-traumatic vasculopathies; however, imaging protocols are not widely used in current clinical practice. Continued technical advancement in acquisition speed, automated analysis, and increased clinician experience may promote its clinical adoption.

### 3.4. Other Methods of Detecting Ischemic Injury

Although CT and MR images with or without angiography are the primary means of diagnosing PTCI, there is emerging interest in other neuromonitoring modalities to detect early post-TBI complications including AIS. Briefly discussed here, transcranial Doppler ultrasound (TCD) and EEG represent non-invasive methods of identifying changes in physiology than can be performed serially throughout the ICU and hospital stay.

#### 3.4.1. Transcranial Doppler Ultrasound

Transcranial Doppler utilizes ultrasound-frequency waves to characterize CBF in real time. It can serve as a useful tool to monitor for VSP after neurologic injury. When used in aSAH, a mean flow velocity (MFV) > 120 cm/s in anterior circulation arteries suggests VSP, while a pulsatility index (PI) > 1.2 indicates poor arterial compliance that can suggest distal VSP or cerebral edema. Its use in TBI is less established and requires further clinical study. A recent meta-analysis showed that similar TCD findings (MFV > 120 cm/s, PI > 1.2) are associated with a 9-fold-increased risk of mortality and greater than 3-fold-increased risk for poor clinical outcome after TBI [71]. Pre-hospital and admission TCD can identify patients with cerebral hypoperfusion after TBI [72,73]; however, less is known about its utility in detecting delayed PTCI. Based on experiences in aSAH and spontaneous AIS, TCD can provide early evidence of VSP that may mediate AIS and can also be used to detect the presence of intracranial occlusion and microembolization secondary to proximal cervical artery vasculopathies such as dissection [74,75]. 

#### 3.4.2. Electroencephalogram

EEG aids in neurodiagnostic assessment by characterizing the electrical activity of the cerebral cortex. The presence of electrographic seizures, epileptiform discharges, and disorganized or slowed waveforms can be indicative of structural lesions such as delayed hemorrhagic expansion or stroke after TBI, but findings may be non-specific. EEG has not been extensively studied in PTCI, but its use in AIS and aSAH suggests a potential role in TBI monitoring. The suppression of all EEG frequencies (termed cortical suppression) has been correlated with a CBF of less than 8–10 mL/100 g/min, and this threshold can be used to detect large vessel stroke [76,77]. Newer continuous EEG modalities, including quantitative EEG, have been proposed as methods for detecting delayed brain ischemia in the ICU [78].

## 4. Management

Serial neuromonitoring with clinical exam and brain imaging is vital to the early detection and management of complications leading to PTCI. A summary of clinical considerations related to diagnosis and treatment are shown in Table 2.

### 4.1. Management of Cerebral Herniation Syndromes

Left untreated, brain herniation leads to irreversible ischemic and mechanical injury and is rapidly fatal. Strategies for early detection and treatment include the implementation of neuromonitoring protocols and the use of stepwise therapy for intracranial hypertension. Early medical treatment is focused on reducing ICP by decreasing intracranial contents including cerebrospinal fluid, cerebral blood volume, and brain water content. The mainstay of medical management for herniation is hyperosmolar therapy with mannitol and hypertonic saline (HTS). Its immediate effect in reversing herniation is targeted at reducing brain water content through the creation of an osmotic gradient that favors water movement from intracellular and interstitial spaces into the vasculature.

There are many practical considerations when using hyperosmolar therapy. Mannitol is commonly used as a first-line therapy, especially outside of a specialized neuroscience ICU, as it is often more readily available and can be easily administered through a peripheral intravenous line. The recommended dose of mannitol is 0.25–1.0 g/kg body weight. Peak effect is seen 15–30 min after infusion, and ICP-lowering effects are expected to last approximately 60 min [79]; however, this is variable in clinical practice. In large or multiple doses, mannitol can cause profound diuresis leading to intravascular volume depletion and hypotension and may not be appropriate in patients in shock or with acute kidney injury [25]. Alternatively, HTS is also used to acutely reverse brain herniation, even if herniation or ICP crises are refractory to mannitol. A study of HTS on herniation reported that 75% of treated patients showed a reversal of transtentorial herniation [80]. Recent evidence shows 23.4% HTS to be effective in reducing ICP by a mean value of 8.3 mmHg when given for ICP > 20 mmHg and increasing CPP by 6 mmHg when pre-treatment values are <70 mmHg [81]. HTS use is also safe and effective in pediatric patients [82]. Bolus doses of 30 mL of 23.4% HTS are typically used. Compared to mannitol, the effects of 23.4% saline appear to be longer-lasting, but the need for a central line for administration limits use in some emergency situations.

Although hyperosmolar therapy is effective in lowering ICP and may reverse clinical herniation, severe TBI may be refractory to medical management. In cases of refractory intracranial hypertension and herniation, surgical DC is considered for definitive treatment. When appropriate, DC in addition to medical management is associated with lower mortality and reduced length of ICU stay [83,84]. Given conflicting studies showing increased mortality following DC, further study is needed to identify the patients most likely to benefit from surgery [85].

In the US, the most cited guidelines for the management of TBI are those proposed by the Brain Trauma Foundation (BTF). In a recent 2020 update, DC is recommended for late or early refractory ICP to reduce ICP and ICU stay, though the mechanistic relationship between DC and favorable outcomes is uncertain [83]. Particularly, the effect of pharmacologic treatment and DC on reducing ischemic injury has not been fully elucidated; however, there is biologic plausibility that improved outcomes are mediated by preventing PTCI associated with herniation. A stepwise approach to intracranial hypertension following TBI is outlined by the latest comprehensive BTF guidelines [86].

### 4.2. Management of Cervical Artery Dissection

Common treatment approaches for CAD include blood pressure control, early anti-thrombotic therapy, and endovascular and open surgery when needed. Antihypertensive medications are key to reducing further arterial wall injury; however, caution needs to be taken if vessel stenosis jeopardizes cerebral perfusion.

The rationale for antithrombotic therapy, including antiplatelet and anticoagulation use, is to prevent the embolization of thrombi from the injured artery and to inhibit the occlusion of stenotic vessels. Currently, there is a paucity of high-quality evidence to compare the efficacies of antiplatelet and anticoagulant drugs for stroke prevention in CAD. A prior systematic review of observational studies suggested no benefit to anticoagulation over antiplatelet use [87]. The Cervical Artery Dissection in Stroke Study (CADISS) was the first multicenter, prospective, randomized controlled trial that compared anticoagulation to antiplatelet treatment in acute symptomatic CAD. The study included patients with extracranial carotid or vertebral artery dissection with an onset of symptoms within the past 7 days. Enrollment consisted of 126 patients assigned to antiplatelet treatment and 124 assigned to anticoagulant treatment. Results showed no difference in rates of ipsilateral stroke between the two groups at 3 months (2% [3/126] vs. 1% [1/124], *p* = 0.63), but the study was underpowered to detect a significant difference in outcome [88]. The study population consisted of mostly spontaneous CAD, which leads to AIS at presumably lower rates than traumatic CAD. 

Based on the scientific evidence, most clinicians treat CAD with antiplatelet drugs as a first-line therapy. Anticoagulation is still used in select high-risk cases, but it is associated with an increased bleeding risk compared to antiplatelet therapy [89]. The rate of recurrent strokes due to traumatic CAD is not well established, but 3–6 months of pharmacologic treatment is suitable in most cases. Treatment duration can be based on follow-up surveillance imaging performed 2–6 months after injury.

Several endovascular options can be considered for those with worsening neurologic symptoms despite antithrombotic therapy. Endovascular interventions have been conducted in the setting of cerebral hypoperfusion distal to a stenotic vessel, in the presence of an expanding intramural hematoma or pseudoaneurysm, or in a worsening dissecting lesion; however, there are no randomized controlled trials to guide treatment decisions. Small clinical studies have demonstrated its efficacy in treating stenosis and pseudoaneurysm formation in populations of spontaneous CAD [90]. Important considerations with endovascular treatment are the increased risk of stroke with catheter manipulation of an acutely injured vessel, peri-procedural iatrogenic arterial dissection, distal thromboembolism, arterial spasm, stent thrombosis, arterial wall perforation, and stent migration [91]. Post-procedural management may include adjunctive dual antiplatelet therapy to prevent stent thrombosis, which may increase the risk of delayed hemorrhagic complications.

Open-surgical treatments are rarely needed for CAD after TBI but can be considered in exceptional cases. Extracranial–intracranial bypass is a revascularization technique performed to preserve cerebral blood flow by circumventing a damaged vessel. Carotid ligation in severe CAD can also be performed when there is adequate collateral cerebral blood flow from the contralateral side. Symptomatic pseudoaneurysms can be directly resected then reconstructed with grafts. Since CAD patients have a relatively low risk of recurrent AIS, endovascular and surgical treatments should be conducted judiciously. Studies have suggested that there is no significant correlation between CAD-induced vascular stenosis or pseudoaneurysm formation and recurrent stroke [92].

Given the relatively rare incidence of traumatic CAD, large, randomized trials to compare efficacy between antiplatelet and anticoagulant therapy may be challenging. Additional research is needed to determine the role of endovascular interventions in more complex cases.

### 4.3. Management of Traumatic Vasospasm

In the absence of high-quality data, managing PTV poses unique challenges. For patients with aSAH, treatment with calcium-channel blockers (CCBs) reduce morbidity, while components of “Triple-H” therapy (hypervolemia, hypertension, and hemodilution) are often initiated to treat VSP. These interventions, however, could be detrimental in the setting of TBI depending on the severity of injury and associated comorbidities. Induced hypertension and hypervolemia can potentially worsen cerebral edema and ICP if cerebral autoregulation is impaired [93]. Additionally, Triple-H therapy may exacerbate non-neurologic traumatic injuries by increasing bleeding risk in polytrauma, resulting in a hypervolemic state and hyperchloremic acidosis [94]. Given these considerations, a reasonable clinical approach is to maintain a euvolemic state while augmenting blood pressure in patients with PTV.

The CCB nimodipine, a standard treatment in aSAH, may also be beneficial in patients with PTV. Small observational studies in TBI have demonstrated improved TCD flow and reduced occurrence of delayed cerebral ischemia and VSP with nimodipine use [95]. Those receiving nimodipine had a significantly lower incidence of death, vegetative survival, or severe disability at 6 months post-trauma; however, larger efficacy studies are needed. Nimodipine may be beneficial in only a select subgroup of TBI patients, particularly in those who develop PTV; however, this has not been confirmed in clinical trials. Undesirable effects of hypotension from CCBs are particularly worrisome in TBI patients as hypoperfusion is an avoidable cause of secondary brain injury and is associated with worse outcomes [35]. 

Other therapies that are used in post-aSAH VSP include intra-arterial papaverine, intra-arterial CCBs such as verapamil, and balloon angioplasty [37]. Microballoon angioplasty in adult aSAH patients was found to significantly lower middle cerebral artery and basilar artery flow velocities [37]. These treatments have also demonstrated reasonable success in case studies, with improvements in clinical outcomes and TCD velocities post-trauma [96]; however, larger studies are needed to determine the optimal treatment of PTV.

### 4.4. Management of Cerebral Venous Thrombosis

Although therapeutic anticoagulation for a period of 3–6 months is the guideline recommendation for non-traumatic CVST to prevent ischemic complications [97], the presence of intracranial hemorrhage or other non-neurologic traumatic injuries often precludes this. The early use of anticoagulation after hematoma stabilization without bleeding complications has been reported in small case series [98]; however, in the absence of high-quality guidance, treatment is often conservative. The prevention of intravascular volume depletion by maintaining euvolemia is thought to prevent clot propagation. Serial venous imaging can be performed in the acute period to monitor for CVST propagation and change treatment strategies if needed.

## 5. Conclusions and Future Research Directions

Despite being a commonly encountered complication after TBI, there remains a lack of clinical understanding of risk factors of PTCI and the best methods for its early diagnosis and management. The pathophysiology of secondary brain injury from ischemia is complex and multifactorial, posing a challenge for future clinical research. The most understood mechanisms of PTCI—namely, brain herniation, CAD, CVST, and PTV—should be studied individually as they may be diagnosed and treated in unique ways. The true incidence of AIS acutely after TBI and its impact on short- and long-term outcomes has not yet been fully established. As evolving research suggests that PTCI significantly impacts mortality and functional outcomes in TBI, the importance of this condition cannot be understated. 

Advanced diagnostic modalities might more accurately describe the prevalence of PTCI. MR techniques such as perfusion imaging potentially would identify areas at risk for irreversible ischemic injury, while diffusion tensor imaging could be used to characterize the impact of TBI and stroke on important neuronal pathways [99]. As discussed, TCD and EEG offer non-invasive methods of neuromonitoring in other acute neurologic diseases and may have applications in moderate-to-severe TBI. Whether increased diagnostic screening is beneficial to patient outcomes and resource utilization is not known. 

The optimal prevention and treatment of PTCI have not been established. Although hyperosmolar therapy is clinically validated to reverse brain herniation, standardized treatments for PTV, CAD, and CVST are lacking in US and international consensus. The goal of clinicians who manage patients with TBI and associated AIS should be the prevention of secondary brain injury. Novel diagnostic and therapeutic approaches are urgently needed to improve outcomes in this population.

## Figures and Tables

**Table 1:** Diagnostic modalities for post-traumatic cerebral infarction and underlying mechanisms of disease.

Diagnostic Modality	Advantages	Limitations
Physical Exam	Allows serial monitoring, no additional cost	Confounded by medication effect and distracting injuries, accuracy dependent on provider experience
Non-contrast CT	Rapid acquisition time	Less sensitive for early and subtle ischemic changes, requires hospital transport, radiation exposure
Non-contrast MRI	Increased sensitivity for early and subtle ischemic changes	Prolonged acquisition time, requires hospital transport, contraindicated with certain medical devices or foreign objects
CT angiography	High-resolution vessel imaging, non-invasive, rapid acquisition time	Contrast may be contraindicated in allergy or acute renal injury
MR angiography	Can be performed with or without contrast enhancement, non-invasive	Prolonged acquisition time, less accurate in low-flow vessels, including small and distal vessels
DSA	High-resolution, real-time vessel imaging	Invasive procedure, not available in all centers
MR VWI	High-resolution vessel imaging, non-invasive	Prolonged acquisition time, not widely available
TCD	Non-invasive study, performed at bedside	Low specificity and sensitivity in diagnosing PTCI
EEG	Non-invasive study, can be monitored continuously, performed at bedside	Low specificity and sensitivity in diagnosing PTCI, confounded by medication effect and metabolic encephalopathies

CT: computed tomography, MRI: magnetic resonance imaging, DSA: digital-subtraction angiography, MR VWI: MR vessel wall imaging, TCD: transcranial Doppler ultrasound, EEG: electroencephalogram, PTCI: post-traumatic cerebral infarction.

**Table 2:** Summary of clinical considerations for common causes of post-traumatic cerebral infarction.

Disease Mechanism	Timing of PTCI	Diagnostic Modality	Treatment Options
Herniation	Delayed	CT, MRI	Hyperosmolar therapy Mannitol 0.25–1.0 g/kg Hypertonic saline bolus Surgical decompression
Arterial Dissection	Early	CTA, MRA DSA MR VWI	Antithrombotic therapy: Antiplatelet drugs Anticoagulation * Endovascular/surgical options
Vasospasm	Delayed	CTA, MRA DSA	Avoidance of hypovolemia Induced hypertension Oral nimodipine Intra-arterial treatment: Calcium-channel blocker Vasodilator Angioplasty
Venous Thrombosis	Early or delayed	CTV, MRV MR VWI	Conservative management Anticoagulation *

* In patients with high risk of post-traumatic cerebral infarction and low risk of hemorrhagic complications. PTCI: post-traumatic cerebral infarction, CT: computed tomography, MRI: magnetic resonance imaging, DSA: digital-subtraction angiography, MR VWI: MR vessel wall imaging, CTV: CT venogram, MRV: MR venogram.

## Data Availability

Data sharing not applicable. No new data were created or analyzed in this study. Data sharing is not applicable to this article.
